# SeptiFast for diagnosis of sepsis in severely ill patients from a Brazilian hospital

**DOI:** 10.1590/S1679-45082014AO2932

**Published:** 2014

**Authors:** Roberta Sitnik, Alexandre Rodrigues Marra, Roberta Cardoso Petroni, Ozires Pereira Santos Ramos, Marinês Dalla Valle Martino, Jacyr Pasternak, Oscar Fernando Pavão dos Santos, Cristóvão Luis Pitangueira Mangueira, João Renato Rebello Pinho

**Affiliations:** 1 Hospital Israelita Albert Einstein, São Paulo, SP, Brazil.

**Keywords:** Sepsis/diagnosis, Multiplex polymerase chain reaction, Real-time polymerase chain reaction

## Abstract

**Objective:**

To test and validate a multiplex real-time polymerase chain reaction method for bloodstream infections, as well as to compare the results with conventional blood culture.

**Methods:**

A total of 114 consecutive patients with clinical evidence of sepsis were submitted to blood culture and LightCycler™ SeptiFast tests.

**Results:**

More positive specimens (23; 20.2%) were detected using the LightCycler™ SeptiFast than the blood culture (17; 14.9%), with an agreement of 86.8%. Discordant results were seen in four patients positive only to blood culture, ten positive only to LightCycler™ SeptiFast and one to different pathogens found by each test. Infections with microorganisms detected only using blood culture reassured the need to perform both tests. The mean time to results for blood culture was 5 days for negative and 3.5 days for positive results. LightCycler™ SeptiFast results were achieved in less than 8 hours.

**Conclusion:**

LightCycler™ SeptiFast showed a high potential as a test to be carried out concomitantly with blood culture for sepsis diagnosis in severely ill patients. This test allowed a faster diagnosis of bacterial and fungal infections that helped to reduce hospital stay and to control the use of antibiotics. LightCycler™ SeptiFast can also eventually detect microorganism and infections that are hardly detected by blood culture, especially *Candida* non-*albicans* infections.

## RESUMO

**Objetivo:**

Testar e validar um método molecular multiplex para detecção de infecções sanguíneas, além de comparar os resultados com os obtidos pela hemocultura convencional.

**Métodos:**

Os testes de hemocultura e o LightCycler^®^ SeptiFast foram realizados em 114 pacientes consecutivos com evidência clínica de sepse.

**Resultados:**

Mais amostras positivas (23; 20,2%) foram detectadas pelo LightCycler^®^ SeptiFast do que pela hemocultura (17; 14,9%), mostrando concordância de 86,8%. Os resultados discordantes foram de quatro pacientes positivos apenas para hemocultura, dez positivos apenas para LightCycler^®^ SeptiFast e um com patógenos diferentes encontrados em cada método. Infecções por micro-organismos não reconhecidos pelo LightCycler^®^ SeptiFast e detectados apelas pela hemocultura confirmam a necessidade da realização dos dois métodos. O tempo médio para os resultados da hemocultura foi de 5 dias para amostras negativas e de 3,5 dias para as positivas. Os resultados pelo LightCycler^®^ SeptiFast foram obtidos em menos de 8 horas.

**Conclusão:**

O LightCycler^®^ SeptiFast mostrou ser um teste de grande potencial para ser realizado simultaneamente à hemocultura para diagnóstico de sepse em doentes graves, permitindo um diagnóstico mais rápido de infecções por bactérias e fungos e, dessa forma, auxiliando a redução do tempo de hospitalização e racionalização do uso de antibióticos. Eventualmente, o LightCycler^®^ SeptiFast pode detectar inclusive infecções por micro-organismos dificilmente detectáveis via hemocultura, especialmente aquelas causadas por *Candida* não *albicans*.

## INTRODUCTION

Sepsis is a leading cause of morbidity and mortality worldwide in hospitalized patients. Studies about sepsis incidence and outcome in Brazil are scarce, but it is considered a major public health problem in intensive care units (ICUs) that causes high costs for health systems.^(1,2)^ There is a great variability in the incidence and mortality of severe sepsis, depending on the method or database used. In the United States, in average, severe sepsis is recorded in 2% of patients admitted to the hospital and annual average increases about 13%.^(3,4)^

Sepsis is caused by a heterogeneous group of infectious etiologies.^(5)^ The early diagnosis and the provision of appropriate treatment are correlated with clinical outcome.^(6–8)^ The early identification of a pathogen increases the chance of targeting the correct etiologic agent and may avoid misuse of antibiotics. Nevertheless, determining the antimicrobial susceptibility of a bacterial isolate is always required for prescription of adequate antimicrobial therapy. Kumar et al.^(9)^ have reported that each hour of delay in effective therapy is associated with a 7.6%-decrease in survival. Conventional blood culture (BC) is the gold standard to detect blood pathogens, but the time required to complete the process can range from 1 to 5 days depending on the organism. Recently, several molecular methods for diagnosis of bloodstream infections were developed, and they are also being used as an adjunct to traditional methods for faster and accurate results.^(10–12)^

Among molecular methods, the first one approved in Brazil by national regulatory agencies was the LightCycler™ SeptiFast v2.0 (LCS) test (Roche Diagnostics, Manheim, Germany). It is an *in vitro* nucleic acid amplification test to detect and identify directly on blood samples 25 common pathogens DNA (bacteria and fungi). These microorganisms are responsible for roughly 90% of all bloodstream infections.^(7,13)^ There are some studies evaluating the diagnostic accuracy and clinical usefulness of LCS, which show that the combination of LCS and BC significantly improve the diagnostic yield, particularly in patients under antibiotic treatment.^(14–17)^

## OBJECTIVE

Considering that rapid pathogen detection may not only facilitates the diagnosis but also provides appropriate and timely therapy, and the few data about this kind of test, particularly in Brazil, the present study tested and validated a multiplex polymerase chain reaction method for bloodstream infections and compared the results obtained with conventional blood culture results.

## METHODS

### Patients

A prospective study was performed involving patients from three different wards of *Hospital Israelita Albert Einstein* (HIAE), São Paulo, Brazil: ICU; emergency room (ER); and oncology patients (ONCO). Patients from the *Hospital Municipal Dr. Moysés Deutsch* (MBOI), located at Jardim Ângela, in the South Peripheral area of São Paulo City, also participated. A total of 114 severely ill patients were enrolled in the study.

The study was conducted in the Molecular Pathology and Microbiology Departments from the Clinical Laboratory from December, 2008 to October 2009. All patients met clinical criteria for sepsis syndrome. Sepsis was defined as an infection plus two or more of the following systemic inflammatory response syndrome criteria: temperature >38°C or <36°C; heart rate >90/min; respiratory rate >20 breaths/min (or carbon dioxide partial pressure – PaCO_2_ <32mmHg); white blood cell count >12,000 cells/*μ*L or <4,000 cells/*μ*L (or >10% band forms).^(18)^

This study was approved by the Institutional Ethical Committee of HIAE (process number 161/2011). No Informed Consent was used because sample collection was part of patients' standard care.

### Multiplex polymerase chain reaction procedure

Polymerase chain reaction (PCR) tests were performed by the Molecular Pathology Department of the Clinical Laboratory at HIAE. Tests were carried out using the LCS and analyzed by the SeptiFast Identification Software (SIS, Roche Diagnostics) by trained staff on molecular methods. This assay amplifies the internal transcribed spacer (ITS) region between the 16S and 23S ribosomal DNA sequences of *Gram*-positive and *Gram*-negative bacteria, and the 18S and 5.8S ribosomal DNA sequence of fungi. ITS region is more specific species than ribosomal RNAs and therefore is best suited for species differentiation by melting curve analysis after amplification using dedicated identification software. Although this is not a quantitative method, concentration is related to the PCR cycle in which the sample became detectable (crossing point - Cp). Low concentrations of coagulase negative *Staphylococci* (CoNS) and *Streptococci*, which reflect the range of workflow contaminations, are not displayed as a positive result.

A single 5mL blood sample was collected from each patient in a sterile EDTA tube along with the first set of BCs. Blood samples were stored at -20°C in the laboratory and multiplex PCR testing were done twice a week, according to manufacturer's instructions. MGrade reagents and plastic ware from Roche Diagnostics were used in all procedure steps to avoid bacterial or fungal contamination.

Strict procedures should be followed to avoid contaminations among samples and from the environmental strains. The lamina flow cabinet used for sample manipulation was extensively wiped with DNA away reagent (Life Technologies, Carlsbad, CA, USA), 70% ethanol and exposed to ultraviolet germicidal lamp for at least 30 minutes just before its use. Precautions also included unidirectional workflow in the laboratory beginning in the pre-amplification area and moving to the post-amplification area. In addition, for sample manipulations, we used longer powder-free gloves; another pair of regular gloves covering sleeves of the lab coat in order to avoid exposure of skin; and dedicated pipettes.

The mechanical lysis of the specimens (3mL of blood) was performed using the SeptiFast Lys Kit and the MagNA Lyser Instrument. After the lysis, specimens were manually extracted with the SeptiFast Prep Kit. Lysed specimens were incubated at high temperature with a protease and chaotropic lysis buffer that releases nucleic acids and protects the released DNA from DNAses in the blood. After one binding and two washes steps, adsorbed nucleic acids were eluted at high temperature. Amplification was conducted on a LightCycler™ Instrument (Roche Molecular Systems) with PCR reagents from LCS. Each run also contained a reagent control, a negative control and an internal control introduced into each specimen along with the lysis reagent. Melting curves were obtained and the SeptiFast identification software v1.0 was used to determine the corresponding melting temperature. The total time for sample extraction and DNA amplification to the final result was roughly 6 to 7 hours.

### Blood culture

Conventional BC was performed in parallel by the Microbiology Department of the laboratory using BACTEC Plus Aerobic/F and BACTEC Plus Anaerobic/F bottles. All bottles were monitored by BACTEC 9240 blood culture system (Becton Dickinson and Company, Flanklin Lakes, New Jersey, USA). When a positive signal was obtained, BC bottles were removed from the instrument, *Gram* staining of the BC medium in the bottles was performed and the results rapidly reported to physicians. Samples were platted onto blood agar, chromogenic agar (chromID™ CPS™ bioMérieux) and anaerobic blood agar. Identification of bacterial or fungal species as well as antibiotic sensitivity tests were then carried out using the Vitek II system (*bioMérieux, Marcy l'Etoile*, France) and API 32 C (*bioMérieux, Marcy l'Etoile*, France) for yeast.

## RESULTS

Sample included 40 (35.1%) women and 74 (64.9%) men with mean age of 49.7 (±24.8) years. Most of the samples were from the ICU (56 samples; 49.1%), but we also received samples from ONCO (38 samples; 33.3%), ER (16 samples; 14.0%) and MBOI (4 samples; 3.5%).

Among the 114 cases, LCS and BC showed positive results in 23 (20.2%) and 17 (14.9%) samples, respectively. A total of 27 cases (23.7%) were positive by one of the two assays (either LCS or BC); some of them showing infections by more than one pathogen (total of 32 detected pathogens). Polymicrobial infections were detected in three patients by the LCS and in another by BC. In one patient, two different pathogens were identified by each method. LCS detected *Klebsiella pneumoniae/oxytoca* while BC detected *Burkholderia cepacia*. This patient had a positive culture result for *K. pneumoniae* in tracheal aspirate. Considering all positive results as true positives, specificity and positive predictive value (PPV) were 100% for both LCS and BC. Sensitivity was 81.3% and negative predictive value (NPV) was 93.5% for LCS while for BC values obtained were 53.6% and 86.1%, respectively. The LCS and BC results for these patients with at least one pathogen detected are shown in table 1.

**Table 1:** Results of LyghtCycler™ System and blood culture in patients with positive results with at least one detection system

Patient		LCS	BC
Positive for both systems (concordant)			
	2	*Klebsiella pneumoniae/oxytoca*	*Klebsiella pneumoniae*
	8	*Pseudomonas aeruginosa*	*Klebsiella pneumoniae; Pseudomonas aeruginosa*
	9	*Pseudomonas aeruginosa*	*Pseudomonas aeruginosa*
	10	*Klebsiella pneumoniae/oxytoca*	*Klebsiella pneumoniae*
	12	*Escherichia coli*	*Escherichia coli*
	13	*Escherichia coli*	*Escherichia coli*
	14	*Escherichia coli*	*Escherichia coli*
	17	*Pseudomonas aeruginosa*	*Pseudomonas aeruginosa*
	18	*Staphylococcus aureus; Pseudomonas aeruginosa*	*Pseudomonas aeruginosa*
	25	*Escherichia coli*	*Escherichia coli*
	26	*Streptococcus pneumoniae; Klebsiella pneumoniae/oxytoca*	*Streptococcus pneumoniae*
	27	*Candida albicans*	*Candida albicans*
Positive for both systems (discordant)			
	11	*Klebsiella pneumoniae/oxytoca*	*Burkholderia cepacia*
Positive only with LCS			
	1	*Pseudomonas aeruginosa*	Negative
	3	*Pseudomonas aeruginosa*	Negative
	4	*Candida tropicalis*	Negative
	6	*Staphylococcus aureus*	Negative
	7	*Pseudomonas aeruginosa*	Negative
	19	*Staphylococcus aureus; Candida albicans; Candida glabrata*	Negative
	20	*Klebsiella pneumoniae/oxytoca*	Negative
	21	*Enterobacter cloacae/aerogenes*	Negative
	22	*Pseudomonas aeruginosa*	Negative
	24	*Pseudomonas aeruginosa*	Negative
Positive only with BC			
	5	Negative	*Staphylococcus epidermidis*
	15	Negative	*Burkholderia cepacia*
	16	Negative	*Burkholderia cepacia*

LCS: LyghtCycler^®^ System; BC: blood culture.

Overall concordance among BC and LCS was 86.8%. Time for BC negative results was 5 days and 3.5 days for positive results. LCS results could be achieved in less than 8 hours.

The isolated BC positive result for *Staphylococcus epidermidis* reflects a software feature that excludes CoNS positive results with Cp values higher than 20 (concentration lower than 100CFU/mL). This reduces the positive rate based on the assumption that they are contaminants and not real causative agents for infection.

For fungi, only one sample was positive for *Candida albicans* using BC, but other three patients were positive for *C. albicans, Candida tropicalis* and *Candida glabrata* using the LCS.

The higher rate of positive results was obtained from ICU patients 28.6%. ER and ONCO patients had a positivity rate of 18.8% and 10.5%, respectively.

Detected pathogens are listed in table 2. *Gram*-negative infections were more frequent and the most common one was the *Pseudomonas aeruginosa*, detected in 7.9% of tested patients.

**Table 2:** Pathogens detected by LightCycler™ SeptiFast and blood culture

	Only PCR	Only HC	PCR and HC
*Gram*-negative bacteria			
*Pseudomonas aeruginosa*	5	0	4
*Klebsiella pneumoniae/oxytoca*	3	1*	2
*Escherichia coli*	0	0	4
*Enterobacter cloacae/aerogenes*	1	0	0
*Gram*-positive bacteria			
*Staphylococcus epidermidis* (CoNS)	0**	2	0
*Staphylococcus aureus*	3	0	0
*Streptococcus pneumoniae*	0	0	1
Fungi			
*Candida albicans*	1	0	1
*Candida tropicalis*	1	0	0
*Candida glabrata*	1	0	0
*Gram*-negative bacteria not detected by SeptiFast			
*Burkholderia cepacia*	ND	3	0

* *Klebsiella pneumonia*;

** considered as a contaminant by SeptiFast if Cp is higher than 20.

PCR: polymerase chain reaction; CoNS: coagulase negative *Staphylococc*; ND: not detected; BC: blood culture.

## DISCUSSION

Results obtained in this study show that LCS is a useful system for rapid diagnosis of sepsis in severely ill patients. The agreement between BC and LCS in our study was 86.8%. Concordant results in previous studies with different kinds of patient populations ranged from 70 to 88%.^(19, 20)^

All *Gram*-negative rods detected by LCS could be real pathogens. Although non-fermentative bacilli, such as *P. aeruginosa*, *Acinetobacter baumannii* and *Stenotrophomonas maltophilia* can be found as environmental contaminants, they are recognized as an important cause of nosocomial infection mainly in immunosuppressed individuals.

CoNS are frequently isolated from blood cultures, in which they may be only a contaminant or the cause of bacteraemia. Despite the careful manipulation of reagents during reaction set up and during extraction up to the real time amplification, considering that human skin and upper respiratory tract are populated with some microorganisms identified by SeptiFast, one could expect a high possibility of CoNS contamination. Indeed, CoNS were detected by BC in two cases. In one of them, CoNS was also detected by LCS with high Cp but was excluded by the LCS software interpretation. This result reinforces the importance of the precautions taken to avoid contamination during all the process, *i.e.*, from sample collection to PCR amplification. Other authors had shown that LCS has a higher positivity rate and a lower contamination rate than BC.^(20, 21)^

Even with BC, determining whether an isolate of CoNS represents a true bacteremia is difficult. García et al.^(22)^ analyzed patients with one or more positive blood culture for CoNS and found a statistically significant difference in the median time to positivity between the clinical bacteremia and contaminations (19.4 *versus* 22.7 hours; p=0.02), showing that time to positivity may be a useful parameter for the diagnosis of true CoNS bacteremia. In the present work, in two patients with positive results for *S. epidermidis* detected only by BC, incubation times to positivity were 22 and 25 hours, which can suggest a possible contamination, especially in the last isolation.

Regarding fungal detection, the conventional blood culture identified only *C. albicans* in one sample. Blood culture system may fail in identifying *Candida* non-*albicans*, as showed by Fernandez et al.^(23)^ These authors also showed that the mean time to positive yeast detection for *C. albicans* was 35.3±18.1 hours, whereas for *C. glabrata* it was 80.0±22.4 hours (p<0.0001). LCS was positive for three *Candida* species: *C. albicans*, *C. tropicalis* and *C. glabrata*. As expected, only the first species was also detected by BC in one of the two positive samples for fungal infections identified by LCS. Fungal pathogen detection was substantially improved with the use of LCS.

Some relevant pathogens were not detected by LCS but only by BC. In our study, *B. cepacia* was detected in 3 patients only by BC.

Discordant results may have different causes. The use of antibiotic before blood sample collection can interfere with culture leading to non-viable microorganism with a LCS positive result. Blood cultures are reported to be negative in about 50% of clinically sepsis cases.^(8)^ On the other hand, a larger volume of blood collected for BC tests or an infection by an organism not included on SeptiFast master list could explain positive BC and negative LCS results.^(24)^

Analyzing the different clinical wards studied, the higher positive rate was observed on the ICU (28.6%), showing clinical utility of the molecular test for this kind of patients. However, in the other wards tested, positive samples were also identified pointing to the impact of implementing the LCS for every patient with suspected sepsis, independently from the clinical ward, provided that they met some pre-established clinical criteria. This is an important point to discuss because clinicians will be able to use a more appropriate antimicrobial therapy for their patients, and as we know this clinical practice is important for decreasing mortality in septic patients.^(25,26)^

Time for result processing is the strongest advantage for using real time PCR. In the present study, because of the need to maintain a separated area of the laboratory and a team dedicated to this reaction, LCS could not be performed at least once a day, that would be needed to keep the turnaround time (TAT) little enough to better evaluate its effects on patient management.

The results obtained were not considered by physicians, since our main aim was to test the feasibility of the LCS in our laboratory and verify its performance characteristics. Ideally, using a team devoted to LCS execution, TAT can be reduced to less than 4 hours, using an automated extraction^(27)^ and its results significantly improved treatment and outcome of patients, even using samples other than blood.^(11,14,16,28)^

The major limitation of LCS is the need of a specialized laboratory that follows strictly guidelines to avoid contaminations from microorganisms, which might be present in the environment, manipulators' skin and secretions. This need is greater than those needs for other nucleic acid amplifications tests that driven to other agents not presented in the environment. Because of LCS high complexity degree, its limitations restrain the use in most clinical routine laboratories. On the other hand, it seems an interesting assay to speed up the identification of microorganisms infections in severely compromised patients. We could show the feasibility of the molecular test in our laboratory that was subject to the compliance with rules to avoid contamination described before LCS validation, which was approved after a detailed study of laboratory workflow in order to avoid environmental contamination and sample-to-sample carryover, as described on the this study method section.

## CONCLUSION

To our knowledge, this is the first study in Brazil using the LightCycler™ SeptiFast methodology. We detected more positive specimens in LightCycler™ SeptiFast than using the blood culture with an overall agreement of 86.8%. Infections by microorganisms that are not identified by SeptiFast were detected only by blood culture, reassuring the need to perform both tests in the routine. Also, LightCycler™ SeptiFast could not detect resistance profile, except the *Staphylococcus aureus* to oxacilin. LightCycler™ SeptiFast showed a high potential as an important test to be carried out concomitantly with blood culture to diagnose patients with suspicion of sepsis. It also allowed a faster diagnosis of bacterial and fungal infections so that reducing hospitalization and antibiotics use. LightCycler™ SeptiFast can also eventually detect some microorganisms infections that are hardly found by blood culture, especially *Candida* non-*albicans* infections.
